# Communicability Characterization of Structural DWI Subcortical Networks in Alzheimer’s Disease

**DOI:** 10.3390/e21050475

**Published:** 2019-05-06

**Authors:** Eufemia Lella, Nicola Amoroso, Domenico Diacono, Angela Lombardi, Tommaso Maggipinto, Alfonso Monaco, Roberto Bellotti, Sabina Tangaro

**Affiliations:** 1 Dipartimento Interateneo di Fisica, Università degli Studi di Bari, 70125 Bari, Italy; 2 Istituto Nazionale di Fisica Nucleare, Sezione di Bari, 70126 Bari, Italy

**Keywords:** brain connectivity, neuroscience, Alzheimer’s disease, diffusion tensor imaging, complex networks, communicability, subcortical brain network

## Abstract

In this paper, we investigate the connectivity alterations of the subcortical brain network due to Alzheimer’s disease (AD). Mostly, the literature investigated AD connectivity abnormalities at the whole brain level or at the cortex level, while very few studies focused on the sub-network composed only by the subcortical regions, especially using diffusion-weighted imaging (DWI) data. In this work, we examine a mixed cohort including 46 healthy controls (HC) and 40 AD patients from the Alzheimer’s Disease Neuroimaging Initiative (ADNI) data set. We reconstruct the brain connectome through the use of state of the art tractography algorithms and we propose a method based on graph communicability to enhance the information content of subcortical brain regions in discriminating AD. We develop a classification framework, achieving 77% of area under the receiver operating characteristic (ROC) curve in the binary discrimination AD vs. HC only using a 12×12 subcortical features matrix. We find some interesting AD-related connectivity patterns highlighting that subcortical regions tend to increase their communicability through cortical regions to compensate the physical connectivity reduction between them due to AD. This study also suggests that AD connectivity alterations mostly regard the inter-connectivity between subcortical and cortical regions rather than the intra-subcortical connectivity.

## 1. Introduction

Complex networks provide a powerful framework to model components of biological systems and their interactions. In particular, they find application in neuroscience research, since they are tailored to study the human brain and its connectivity. In fact, the brain can be modeled as a system with several regions, whose interactions produce complex behaviors [1].

The reconstruction of the brain connectivity network can be achieved through the use of magnetic resonance imaging (MRI) techniques, including structural MRI (sMRI), functional MRI (fMRI) and diffusion-weighted imaging (DWI). A large number of studies (e.g., [2,3,4,5,6]) have been conducted to examine anatomical and functional connectivity in order to understand the organization and topology of the brain network and for identifying critical areas. In particular, several studies (e.g., [7]) focused on the brain connectivity alterations due to neurodegenerative diseases to understand the mechanisms underlying such degeneration.

Among neurodegenerative brain disorders, Alzheimer’s disease (AD) is the most widespread, characterized by a progressive deterioration of cognitive and memory functions [8]. There is a growing body of evidence, e.g., [9,10], suggesting that this decline is related to a disrupted connectivity among brain regions, due to white matter (WM) degeneration. DWI is particularly suited to investigate the integrity of WM fiber tracts by measuring the water diffusion along them. Pulsed magnetic field gradients are applied along the three axes x,y,z. The strength of the $b_{0}$ field is linearly altered and this makes the magnetic resonance signal sensitive to the diffusion coefficient. For each voxel, a diffusion tensor is defined describing the molecular mobility along the gradient axes and the correlation between these directions [11]. Starting from DWI scans, and using tractography algorithms together with a parcellation scheme, anatomical brain connectivity networks can be constructed. Some fiber tracking algorithms use the diffusion tensor to track fibers along their whole length. In this case, for each brain voxel, the dominant direction of axonal tracts can be assumed to be parallel to the primary eigenvector of the diffusion tensor. Thus, these fiber tracking algorithms use the diffusion tensor of each voxel to follow an axonal tract in 3D from voxel to voxel. In case of crossing fibers, the assumption that in each voxel there is a unique orientation of the fibers is not valid and higher order models, such as new tractography algorithms based on constrained spherical deconvolution, have been developed in order to address this issue [12]. By investigating the tractography networks, AD-induced abnormalities in the topological organization of the brain have been found [13] and AD-related group differences of network metrics were found also at preclinical stages [14].

Recently, we successfully used the communicability metric, introduced in [15], to uncover connectivity differences in AD brain networks reconstructed at the cortical level and we developed a graph-based classification framework to distinguish unhealthy from healthy subjects automatically [16]. Since communicability quantifies the ease of communication between two nodes, taking into account not only the shortest path but all possible paths connecting them, it revealed to be particularly suited to describe the alteration of communication between brain regions due to AD, and it outperformed more classic network measures when used as feature of a supervised classification algorithm. In fact, this metric provides a more global measure of the efficiency of the information spread through a network, and so it can be more useful than other network measures to characterize the brain connectivity alterations due to a disconnection syndrome related to an integrity loss of the communication routes.

It should be emphasized that most of the existing literature in this context focused on AD connectivity abnormalities at a relatively global level, such as the whole-brain level [17], and at the cortex level, as in our previous study [16] and in [13,18]. There has been much less research working on understanding the AD-related network changes at the subcortical structural level. The study of the subcortical connectivity is really important for the AD characterization because of the key role played by these regions in triggering the disease [19]. Furthermore, to the best of our knowledge, no subcortical connectivity network study has been conducted on DWI data but only on structural MRI and fMRI, as in [20]. Our hypothesis is that extracting the subcortical sub-network from the whole communicability network, instead of extracting the subcortical sub-network from the whole weight network, could enhance the information content of subcortical regions in discriminating AD and in revealing AD-related subcortical connectivity patterns. In this work, we analyze the subcortical structural connectivity networks of 46 healthy control (HC) subjects and 40 AD patients. We measure the information content of subcortical connections in discriminating AD from normal subjects, in terms of communicability between the subcortical regions and between these regions and the rest of the network. This investigation is carried out both with a statistical-descriptive approach and from a machine learning perspective. We show that communicability is able to highlight interesting subcortical connectivity patterns in AD, which do not emerge from simply considering the connectome. We also show that, when using graph measures involving only the subcortical regions as features for AD discrimination, communicability values are significantly better then graph weights.

## 2. Materials

In this study, we employed data from the Alzheimer’s Disease Neuroimaging Initiative (ADNI) database (http://adni.loni.usc.edu/). ADNI was launched in 2003 as a multisite, longitudinal study, which combines several biological markers and clinical and neuropsychological assessments to measure the progression of early AD. Determination of sensitive and specific markers of AD progression is intended to aid researchers and clinicians to develop new treatments and monitor their effectiveness, as well as to lessen the time and cost of clinical trials.

The scans processed for the present analysis belong to 86 subjects, 46 HC subjects and 40 AD patients. HC subjects show no sign of depression, mild cognitive impairment or dementia; participants with AD are those who meet the NINCDS/ADRDA criteria for probable AD. Each subject underwent a cognitive evaluation including the mini-mental state examination test (MMSE) (scores less than 24 out of 30 indicate impairment) [21] and Alzheimer’s disease assessment scale (ADAS) (scores less than or equal to 10 may be considered in the normal range) [22]. Demographics and clinical scores for the participants are summarized in Table 1. The diffusion-weighted scans were acquired using a 3T GE Medical Systems scanner. For each subject, we considered both T1-weighted 3D anatomical spoiled gradient echo (SPGR) sequences (256 × 256 matrix; voxel size = 1.2×1.0×1.0 mm${}^{3}$; TI = 400 ms; TR = 6.98 ms; TE = 2.85 ms; flip angle = $11^{\circ }$), and diffusion weighted images (256 × 256 matrix with a field of view of 35 cm; voxel size = 2.7 × 2.7 × 2.7 mm${}^{3}$; scan time = 9 min; repetition time/echo time = 9 s/60 ms; flip angle = $90^{\circ }$). More precisely, concerning DWI, 46 separate images were acquired for each scan: 5 with negligible diffusion effects ($b_{0}$ images) and 41 diffusion-weighted images (b=1000 s/mm${}^{2}$). More details can be found at http://adni.loni.usc.edu/wp-content/uploads/2010/05/ADNI2GE3T22.0T2.pdf.

## 3. Methods

The analysis we carried out consisted of a number of steps which are described in the following subsections. It is worth to note that all data analyses here presented require huge computational capacities, with image processing time, in particular, of more than ten hours per subject. For this reason, we used the distributed infrastructure in the ReCaS-Bari computing farm https://www.recas-bari.it.

### 3.1. Image Processing

**Figure 1:**
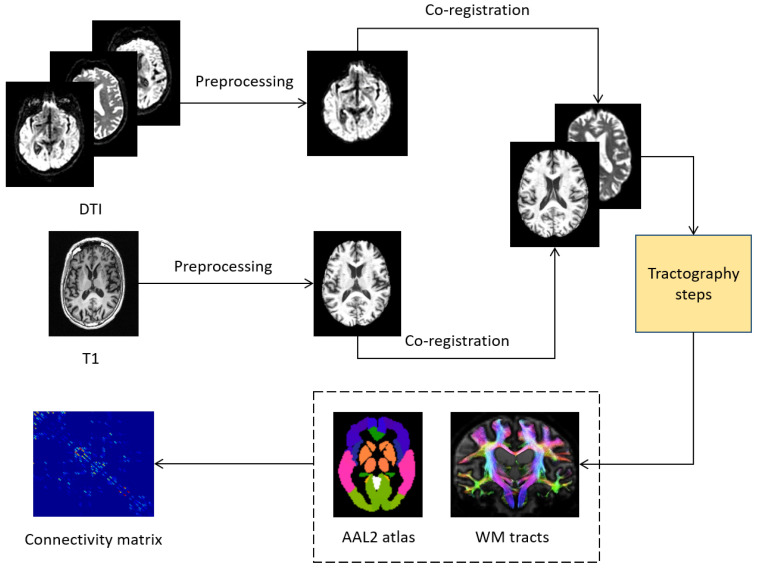
Main steps of the image processing pipeline.

The image processing steps we performed allow for the reconstruction of the brain connectome from the raw DWI scans. For each subject, the raw DICOM images were acquired from the ADNI database. The dcm2nii software, within the MRIcron suite, was used to convert the DICOM images into the NIFTI format. The NIFTI images were then organized in the standard BIDS format. The subsequent processing steps, from image preprocessing to structural connectome generation, were performed using tools provided by the MRtrix3 software package http://mrtrix.org [24,25], including, for some preprocessing step, scripts interfacing with the external package FSL FMRIB Software Library (FSL) https://fsl.fmrib.ox.ac.uk/fsl/fslwiki/ [26]. The main steps of the whole procedure are depicted in Figure 1. Diffusion-weighted images were preprocessed following some standard steps. First of all, a denoising step was performed to enhance the signal-to-noise ratio (SNR) of the diffusion-weighted MR signals in order to reduce thermal noise, due to the stochastic thermal motion of water molecules and their interaction with surrounding microstructure, and which propagates to the diffusion parameters of interest [27]. Head motion and eddy current distortion in each subject were corrected by aligning the DWI images to the average $b_{0}$ image with FSL’s eddy correct tool. The brain extraction tool (BET) [28] was then used to skull-strip the brains. Then the bias-field correction was performed by first estimating a correction field from the $b_{0}$ image, then applying the field to correct all diffusion-weighted volumes. The T1-weighted anatomical scans were preprocessed with the fsl_anat tool performing the following stages: reorientation to the standard image MNI152, automatic cropping, bias-field correction, registration to the linear and non-linear standard space, brain-extraction. The next step was the inter-modal registration for each subject between the T1-weighted anatomical image and the diffusion-weighted image.

After the preprocessing and co-registration steps, a structural connectome generation pipeline was performed. First, a tissue-segmented image appropriate for anatomically constrained tractography was generated [29] (MRtrix command 5ttgen). Then, unsupervised estimation of WM, gray matter and cerebro-spinal fluid response functions was performed (MRtrix command dwi2response dhollander). In the next step, the fibre orientation distributions (FOD) for spherical deconvolution [30] was estimated (MRtrix command dwi2fod msmt_csd). The following step was the probabilistic tractography [31] utilizing dynamic seeding [32] and anatomically-constrained tractography (ACT) [33], which improves tractography reconstruction using anatomical information through a dynamic thresholding strategy. The initial tractogram was generated (MRtrix command tckgen, with 14 million streamlines, maximum tract length = 250, FA cutoff = 0.06) and the Spherical-deconvolution Informed Filtering of Tractograms (SIFT2) [32] methodology was applied (MRtrix command tcksift2). SIFT2 not only provides more biologically meaningful estimates of structural connection density, but also allows for a more computationally efficient solution to the streamlines connectivity quantification problem: by determining an appropriate cross-sectional area multiplier for each streamline rather than removing streamlines altogether, biologically accurate measures of fibre connectivity were obtained whilst making use of the complete streamlines reconstruction [32]. The obtained streamlines were then mapped through a T1 parcellation scheme using the AAL2 atlas [34] (120 regions), a revised version of the automated anatomical atlas (AAL). The last step was a robust structural connectome construction [35] (MRtrix command tck2connectome) that generates a connectome matrix from the streamlines file and the parcellation scheme. This pipeline is used in other recent structural connectivity studies performing tractography (e.g., [36,37]).

### 3.2. Subcortical Network Analysis

The output of the image processing steps was a 120×120 weighted symmetric connectivity matrix *W* for each subject, whose entries $w_{ij}$ corresponded to the number of fiber tracts connecting region *i* to region *j*. The resulting matrices were quite sparse (the mean density was 0.42±0.06 for HC and 0.40±0.06 for AD). In order to evaluate the consistency across participants in the two diagnostic groups, the correlation between the upper triangular matrices was calculated for each pair of subjects: we found a mean correlation of 0.83±0.13 for the HC group and 0.83±0.14 for the AD group. The matrices were normalized in the range $\left[0,1\right]$ by dividing all weights of each matrix by the maximum weight. For each subject, the subcortical sub-network was extracted, including Hippocampus, Amygdala, Caudate, Putamen, Pallidum and Thalamus, both right and left, resulting in a 12×12 subcortical weighted connectivity matrix $W^{sub}$.

In order to investigate the connectivity changes due to AD at the subcortical level, we analyzed the role of the subcortical regions in distinguishing the two groups under observation in terms of communicability.

As a first step, we calculated the communicability between pairs of subcortical regions, considering only the subcortical network $W^{sub}$. Based on the definition provided in [38], the *weighted communicability* for each node pair *p* and *q* of the subcortical network $W^{sub}$ was calculated as follows:

(1) $${\left[G\left(W^{sub}\right)\right]}_{pq}=\sum_{k=0}^{\infty }\frac{{\left(M^{k}\right)}_{pq}}{k!}={\left(e^{M}\right)}_{pq},$$

where $M=D^{-1/2}W^{sub}D^{-1/2}$, being *D* the diagonal subcortical strength matrix.

Generally for a weighted network described by the weight matrix {$w_{ij}$}, the strength of node *i* is defined as the sum of the weights of all edges attached to node *i*:

(2) $$S_{i}=\sum_{j\in N}w_{ij},$$

where *N* is the set of nodes of the network. Thus, from the $G(W^{sub})$ matrix, we can define the intra-strength communicability for each node $i\in N_{s}$, being $N_{s}$ the set of nodes of the subcortical network:

(3) $${SC_{i}}^{intra}=\sum_{j\in N_{s}}{\left[G\left(W^{sub}\right)\right]}_{ij},$$

which expresses the intensity of the total node connectivity with the other subcortical regions, in terms of communicability.

Then, in order to evaluate the extent of the inter-communication between the subcortical regions and the rest of the network, we calculated the whole communicability matrix G(W), whose elements are the communicability values between the node pairs of the whole network, i.e., the network including both cortical and sub-cortical regions. From this matrix, we defined the inter-strength communicability of a subcortical node *i* as the communicability between *i* and only the cortical nodes. Being *N* the set of the whole matrix indices and *S* the subset of the whole matrix indices corresponding to the subcortical regions, ∀i∈S the inter-strength communicability can be defined as:

(4) $${SC_{i}}^{inter}=\sum_{j\in \left\{N-\left\{S-i\right\}\right\}}{\left[G\left(W\right)\right]}_{ij},$$

which expresses the intensity of the total subcortical node connectivity, in terms of communicability, with the rest of the whole network, excluding the other subcortical regions.

**Figure 2:**
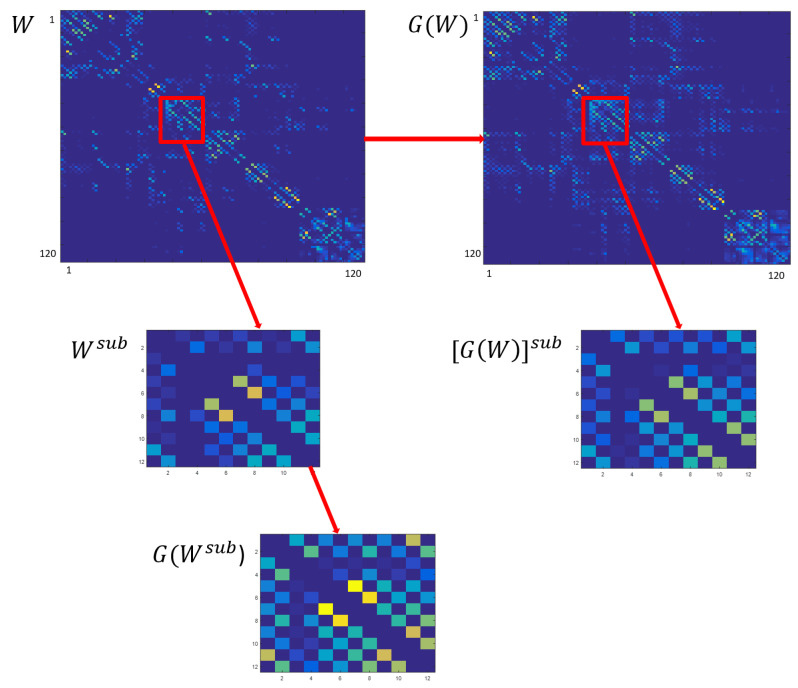
Representative scheme of the construction of the three matrices $W^{sub}$, $G(W^{sub})$ and ${\left[G\left(W\right)\right]}^{sub}$ starting from the whole connectivity matrix *W*.

Additionally, starting from the whole communicability matrix G(W), we extracted the communicability sub-network considering only the entries corresponding to the subcortical regions: we obtained a subcortical network whose entry (i,j) is the communicability value between two subcortical regions which, in this case, takes into account also the possible path between *i* and *j* passing through cortical nodes. We called this matrix the extracted sub-network communicability matrix, ${\left[G\left(W\right)\right]}^{sub}$. Therefore, for each subject we had three matrices describing the subcortical network, calculated starting from the whole connectivity matrix: $W^{sub}$, $G(W^{sub})$ and ${\left[G\left(W\right)\right]}^{sub}$ (Figure 2).

### 3.3. Group-Wise Statistical Analysis

For all subjects, the weighted sub-network communicability ${\left[G\left(W^{sub}\right)\right]}_{pq}$ and the extracted sub-network communicability ${\left[G\left(W\right)\right]}_{pq}^{sub}$ were calculated for each node pair pq. Thus each subject was characterized by three sets of features, i.e., the entries of the three matrices $W^{sub}$, $G(W^{sub})$ and ${\left[G\left(W\right)\right]}^{sub}$. For each set of features, a group-wise statistical analysis was performed in order to identify subcortical brain region pairs with statistically significant difference between HC and AD. In order to make the identification of the significantly different brain regions robust, permutation tests were performed by randomly assigning subjects to the two comparison groups 10,000 times. Differences were considered significant if they did not belong to 95% of the null distribution derived from the permutation tests (corrected *p*-value < 0.05). The False Discovery Rate [39] was used for multiple comparison correction.

The same statistical analysis was performed in order to find if any of the brain regions, within the 12 subcortical regions, had a statistically different intra-strength communicability $SC^{intra}$, or a statistically different inter-strength communicability $SC^{inter}$ between the two groups HC and AD.

### 3.4. Classification

The second goal of the present work was to evaluate the discriminating power of the subcortical regions’ communicability in distinguishing between HC and AD from a machine learning perspective. To this end, a supervised classification framework has been developed based on the random forest (RF) classifier.

Since the dataset is small, the classification procedure has been validated through a 50-times repeated 10-fold cross-validation. As features to feed the classification model, we considered the three sets of features, i.e., the entries of the three matrices $W^{sub}$, $G(W^{sub})$ and ${\left[G\left(W\right)\right]}^{sub}$ calculated from the connectivity matrices obtained by the image processing steps described in Section 3.1.

The folds were stratified by diagnosis to have approximately the same number of subjects from each diagnostic group in each fold. Within each cross-validation iteration, the training set was subjected to two feature selection steps in order to select the most relevant features. Note that a nested feature selection within the cross-validation helps avoid a feature selection bias which may lead to overoptimistic results [40,41].

The first step was an ad hoc selection of features, customized to connectivity networks, already used in [16]. An average matrix was calculated from the HC subjects of each training set, resulting in a weighted matrix whose entries $e_{ij}$ range from 0 to 1 and represent the frequency at which the corresponding edges occur among the HC matrices. This matrix has then been thresholded (the threshold in this case turned to be 0.6, using the binomial test with α=0.01), obtaining a binary matrix to be used as a mask. The matrices of all subjects were then projected onto this mask to select the features to be considered in the following step. The second step was a more conventional recursive feature elimination (RFE) based on support vector machines (SVMs). Briefly speaking, SVMs are a classification model which works by constructing a separating hyperplane between the two classes, so that the minimal distance from the closest data points of either classes is the largest [42]. RFE uses criteria derived from the SVM model to assess feature importance and removes features having small criteria. The process is iteratively computed until all features have been removed and the final output is a ranked feature list. Feature selection is done by choosing a set of top-ranked features [43].

Finally, a RF classifier was trained on the selected features. RF is a state-of-the-art ensemble method for classification which builds a “forest” of decision trees at training time and outputs the mode of the classes predicted by each individual tree at test time [44]. More precisely, RF repeatedly (*B* times) selected a random sample with replacement from the training set and fits a decision tree to this sample based on a subset of randomly selected features. The final predictions of unseen test examples were obtained via majority voting of the *B* single predictions. *B* is a free parameter: in the present work, we set B=500 trees.

## 4. Results

### 4.1. Group-Wise Statistical Analysis

**Figure 3:**
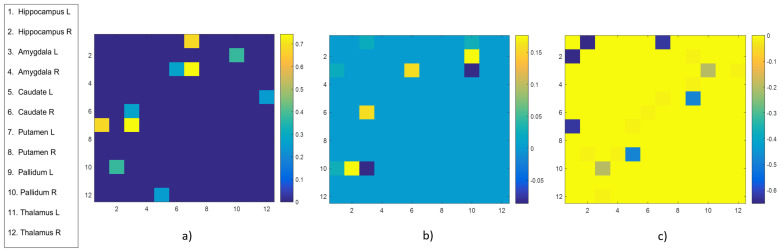
Heat map visualization of the relative differences between the mean values of the significant region pairs in the healthy controls (HC) and AD group for the three cases: (**a**) $W^{sub}$, (**b**) $G(W^{sub})$ and (**c**) ${\left[G\left(W\right)\right]}^{sub}$. The edge color is descriptive of the values.

In order to investigate changes in physical connectivity (edge weights) and in communicability, subcortical brain region pairs with significant (*p*-value < 0.05) group-wise differences were identified considering the three matrices $W^{sub}$, $G(W^{sub})$ and ${\left[G\left(W\right)\right]}^{sub}$. In particular, considering the weights matrix, five of 78 subcortical region pairs were found: (left Hippocampus, left Putamen), (right Hippocampus, right Pallidum), (left Amygdala, right Caudate), (left Amygdala, left Putamen), (left Caudate, right Thalamus). Figure 3a shows the relative difference between the HC mean weight and the AD mean weight for the five identified node pairs normalized by the HC mean weight. It can be observed an average disruption of subcortical physical connectivity in AD subjects for all region pairs.

Also in the case of the sub-network communicability $G(W^{sub})$, five of 78 subcortical region pairs were found to have significantly different values: (left Hippocampus, left Amygdala), (left Hippocampus, right Pallidum), (right Hippocampus, right Pallidum), (left Amygdala, right Caudate), (left Amygdala, right Pallidum). Figure 3b shows the relative difference between the HC mean subcortical communicability and the AD mean subcortical communicability for the five identified node pairs normalized by the HC mean communicability. An average disruption of subcortical communicability in AD subjects can be observed for all region pairs except for (left Amygdala, right Pallidum). Figure 3c shows the same results when considering the extracted sub-network communicability ${\left[G\left(W\right)\right]}^{sub}$. In this case, we found nine of 78 subcortical region pairs with significantly different communicability for the two groups: (left Hippocampus, right Hippocampus), (left Hippocampus, left Putamen), (right Hippocampus, left Pallidum), (left Amygdala, Right Pallidum), (left Amygdala, right Thalamus), (right Amygdala, right Pallidum), (left Caudate, left Putamen), (left Caudate, left Pallidum), (right Caudate, right Caudate). Each significant region pair shows, in this case, an increased communicability in AD compared to HC.

**Figure 4:**
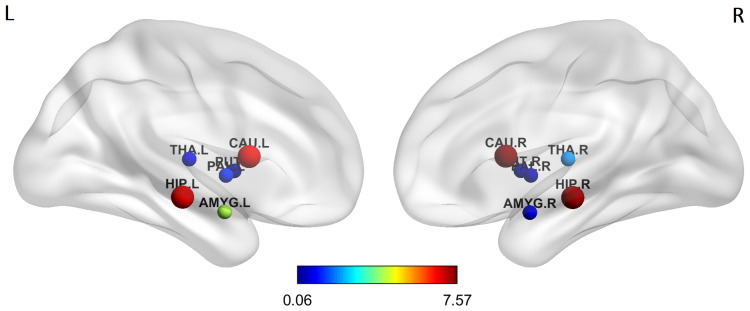
Medial view of left and right hemispheres which shows the group-wise difference of mean inter strength communicability (absolute value in percentage). Information about the absolute value of inter strength communicability of each regions is coded by the color of the ROI node, while statistically significant regions are marked with bigger node sizes. Putamen (both left and right) exhibits mean value greater in AD than in HC.

Concerning the intra strength communicability $SC^{intra}$, no statistically significant difference between HC and AD was found. Instead, the permutation test revealed that in terms of inter strength communicability $SC^{inter}$ the regions with statistically difference at the 0.05 significance level are left and right Hippocampus and left and right Caudate (*p*-value = 0.005 for left Hippocampus, *p*-value = 0.01 for right Hippocampus, *p*-value = 0.001 for left Caudate, *p*-value < 0.001 for right Caudate in the permutation test). The absolute value of the relative difference between the mean HC inter strength communicability and the mean AD inter strength communicability for each subcortical region is depicted in Figure 4 and is expressed in percentage: regions with significant group-wise difference are marked with bigger node sizes.

### 4.2. Classification

Following the procedure described in Section 3.4, the mean classification performance, averaged over all the cross-validation iterations, were measured in terms of accuracy, area under the receiver operating characteristic (ROC) curve (AUC), sensitivity and specificity considering the three sets of features. These performances quantify how well the three measures $W^{sub}$, $G(W^{sub})$ and ${\left[G\left(W\right)\right]}^{sub}$, calculated between only the subcortical regions, discriminate between AD patients and HC subjects.

**Figure 5:**
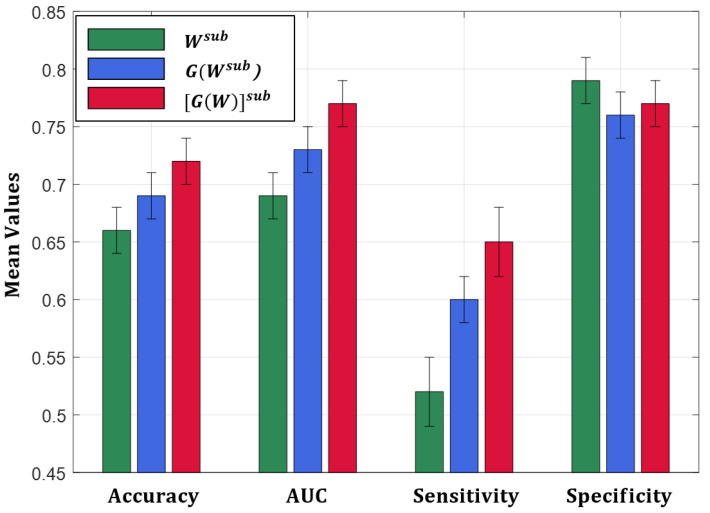
Classification performance comparison.

The results, depicted in Figure 5, show a comparison between the performance obtained in the three cases, thus allowing us to estimate the different information content of the three measures, calculated on the subcortical region pairs, in distinguish between HC and AD.

The best classification performance in terms of accuracy, AUC and sensitivity (0.72±0.02 of accuracy, 0.77±0.02 of AUC and 0.65±0.03 of sensitivity) are obtained using ${\left[G\left(W\right)\right]}^{sub}$. The best mean specificity (0.79±0.02) is reached using weights but it corresponds to a very law value of sensitivity (0.53±0.03). Therefore, globally, ${\left[G\left(W\right)\right]}^{sub}$ provides the best performance.

## 5. Discussion and Conclusions

The aim of this work was to study the information content of the subcortical DWI sub-network in discriminating AD from HC subjects and to evaluate the usefulness of the communicability metric to describe the subcortical connectivity and its alterations caused by AD. To the best of our knowledge, there has been little research working on understanding the AD-related network changes at the subcortical structural level, especially on DWI data. The present analysis took a step in this direction. Moreover, in this work the communicability metric has been used for the first time to identify the connectivity profile of the subcortical network. The information content of the subcortical network for the HC/AD discrimination was investigated in terms of edge weights and in terms of communicability. In particular, two types of sub-network communicability were defined: the sub-network communicability $G(W^{sub})$ calculated starting from the subcortical weights matrix, and the extracted sub-network communicability ${\left[G\left(W\right)\right]}^{sub}$, extracted from the whole communicability matrix. The analysis was conducted from two points of view:

(i) A group-wise statistical analysis has been performed to find subcortical brain region pairs with significantly different values of weights and communicability. The same analysis has been conducted to identify subcortical regions with different intra and inter-strength communicability, that are measures introduced to quantify the total intensity of subcortical nodes’ connectivity, in terms of communicability with the other subcortical nodes and with the rest of the whole network.
(ii) A classification procedure has been adopted to investigate to which extent the sub-network communicability values and the extracted sub-network communicability between the subcortical regions are able to automatically discriminate between HC subjects and AD patients. The performance were also compared to the ones obtained using the subcortical edge weights as features for training the classification models.

Concerning the group-wise statistical analysis on the subcortical edge weights, five subcortical region pairs have shown statistically significant different values between HC and AD, mostly involving Hippocampus, Amygdala, Putamen and Caudate. The key role played by these regions in AD is well known [45,46,47,48,49,50]. It is worth noting that an average connectivity degradation emerges in AD patients when considering the physical connections between the subcortical regions, as it could be expected.

Regarding the group-wise statistical analysis on the subcortical node pairs communicability, calculated starting from the subcortical weight sub-network $G(W^{sub})$, five subcortical region pairs were found with significantly different communicability between HC and AD, mostly involving Hippocampus and Amygdala, but also Pallidum and Caudate. It is worth noting that, also in this case, an average communicability disruption is observed in AD but, in spite of this, the region pair (left Amygdala, right Pallidum) shows an increased communicability in AD compared to HC. Two significantly different region pairs were in common with the findings previously obtained from the weights matrix: (right Hippocampus, right Pallidum) and (left Amygdala, right Caudate). Thus, using the sub-network communicability matrix $G(W^{sub})$, instead of the weights matrix $W^{sub}$, for the characterization of the subcortical connectivity alterations in the patological scenario, seems to not provide additional relevant information.

Different conclusions can be reached considering the group-wise statistical analysis on the extracted sub-network communicability ${\left[G\left(W\right)\right]}^{sub}$. First of all, nine significant node pairs were found and the mostly involved regions are again Hippocampus, Amygdala, Caudate and Pallidum. In particular the node pairs (left Caudate, left Putamen) and (left Caudate, left Pallidum) were recently found among the top-ranked AD predictor variables in connectome DWI networks in a study considering the whole brain graph [51]. Compared to the previous cases, it is worth noting that in this case all significantly region pairs showed a greater extracted communicability in AD compared to HC. This is not the first time some regions with greater communicability in patients compared to controls have been reported in a disconnection syndrome and using DWI data. In [52], some areas of greater communicability in stroke patients compared to controls were found also in the lesioned hemisphere. One possible interpretation of these results may be that the increased communicability in AD reflects adaptive changes in the white matter structure that have occurred secondary to the disease. It is interesting to underline that communicability is not limited to merely consider the physical connections between two nodes, like weights do, or to consider the shortest path lenght between them, but takes into account all possible paths connecting them passing, in this case, also through the cortical nodes. It may be hypothesized that the subcortical regions intensify the communication along the possible routes connecting them in order to compensate the average reduction of physical connectivity occurring between them because of AD. In particular, if we look at the pair (left Hippocampus, left Putamen), it shows a significant reduction of weight in AD but a significant increase of extracted communicability.

Another interesting result concerns the group-wise comparison of inter-strength communicability. Hippocampus (both right and left) again plays an important role showing a statistical significant difference, between HC and AD, together with the left and right Caudate. These results are in line with the literature, in particular with the evidence that one of the first events in AD is the disconnection of the hippocampal formation and neocortex [53]; moreover, neuropathological studies have documented the isolation of the hippocampal formation in AD, e.g., [9]. The connectivity patterns regarding the inter-strength communicability of Hippocampus and Caudate should be more in-depth investigated analyzing their single connections with each cortical region.

Conversely, no statistically significant difference between HC and AD was found considering the intra-strength communicability $SC^{intra}$. These results suggest that the connectivity changes in AD mostly involve the connections between subcortical and cortical regions or between subcortical regions through cortical regions.

The same conclusions can be reached considering the classification comparison. The best classification performance, in fact, are obtained using as features the extracted sub-network communicability (0.72±0.02 of accuracy, 0.77±0.02 of AUC, 0.77±0.02 of specificity and 0.65±0.03 of sensitivity). This comparison suggests that, when using subcortical network-based features, the communicability between brain regions is more informative then weights for AD discrimination, especially when considering all possible communication routes between subcortical brain regions passing through cortical nodes. It is worth to note that the best performance obtained, using only the 12×12 subcortical network, are quite comparable to those we obtained with the 96×96 cortical network in our previous study [16] and in other studies conducted on the ADNI database using network measures extracted from the whole brain network [54] and from the cortical brain network [55]. Future work should address how to better combine cortical and subcortical regions to develop more accurate classification models. Moreover, because of the complex fibre layout in subcortical regions, DWI reliability in subcortical connections reconstruction could be affected by the dataset quality and by the choise of the parcellation scheme. Thus, further analysis will be performed to investigate the informative power of complex patterns of the WM fibers by adopting more atlases and different public datasets, e.g., NACC [56].

The results presented in this work bring to the following conclusions:

(i) The weights of brain networks, which have widespread use in literature to describe the brain connectivity, could not be informative enough, taken alone, to discriminate between HC and AD when relying on the subcortical regions’ connectivity.
(ii) If the whole brain network communicability matrix is calculated and a sub-network communicability is extracted including the 12 subcortical regions (which are well-known AD related-brain regions), these features describe AD connectivity changes better than subcortical edge weights, and lead to better classification performance.
(iii) Using the communicability metric gives a different viewpoint to describe the subcortical brain connectivity and allowed us to point out a sort of resilience mechanism of subcortical regions that tends to increase their communication (mainly through cortical nodes) in order to compensate the physical structural disconnection occurring between them because of AD.

It would be appropriate to introduce an innovative connectivity model based on communicability to perform diverse connectivity analyses that could uncover AD-related alterations difficult to be observed by simply considering the connectome. We believe that this model may be generalized and applied to investigate various diseases related to connectivity aberrations and disconnection syndromes, such as Parkinson’s disease [57].

## Figures and Tables

**Table 1:** Demographic and clinical characteristics of the study participants. For the clinical assessment, the mini-mental state examination test (MMSE), Alzheimer’s disease assessment scale (ADAS) 11 [22] and ADAS 13 [23] scores are reported. According to the t-test statistics, MMSE, ADAS 11 and ADAS 13 are significantly different between healthy controls (HC) and Alzheimer’s disease (AD). For age and gender, the chi-squared test was performed.

	HC (46)	AD (40)	*p*-Value
**Age**	73.2±5.71	74.78±8.37	0.31
**Gender**	21 M/25 F	25 M/15 F	0.11
**MMSE**	29.02±1.17	23.43±1.81	<0.0001
**ADAS 11**	5.64±3.26	20.32±7.13	<0.0001
**ADAS 13**	9.08±4.76	30.44±8.24	<0.0001
